# Supplementary material for: New Insights on Saporin Resistance to Chemical Derivatization with Heterobifunctional Reagents
Source: Biomedicines. 2023 Apr 19;11(4):1214. doi: 10.3390/biomedicines11041214 (PMC10135674; doi:10.3390/biomedicines11041214)
Supplement: Supplementary file 1 [file biomedicines-11-01214-s001.zip › biomedicines-2330980-supplementary.pdf]

Table S1. Poisson probability of each number of -SH groups inserted for the different derivatization -SH/RIP ratios.

		mean derivatization -SH/RIP ratio		
		**1.71**	**1.98**	**2.52**
	**-SH inserted**	**Discrete probability (%)[1]**		
**2-IT**	0	18.1	13.8	8.1
	1	30.9	27.3	20.3
	2	26.4	27.1	25.6
	3	15.1	17.9	21.5
	4	6.4	8.8	13.5
	5	2.2	3.5	6.8
		**mean derivatization-SH/RIP ratio**		
		**1.91**	**2.23**	**3.02**
	**-SH inserted**	**Discrete probability (%)**		
**SPDP**	0	14.8	10.8	4.9
	1	28.3	24.0	14.7
	2	27.0	26.7	22.3
	3	17.2	19.9	22.4
	4	8.2	11.1	16.9
	5	3.1	4.9	10.2
		**mean derivatization -SH/RIP ratio**		
		**1.20**	**1.45**	**1.60**
	**-SH inserted**	**Discrete probability (%)**		
**SMPT**	0	30.1	23.5	20.2
	1	36.1	34.0	32.3
	2	21.7	24.7	25.8
	3	8.7	11.9	13.8
	4	2.6	4.3	5.5
	5	0.6	1.3	1.8

[1] The probability, that a given number of -SH groups are inserted per RIP molecule in a mixture having a given mean derivatization -SH/RIP ratio, was calculated using the Poisson distribution. The probability was calculated for the three mean derivatization ratios obtained with 2-IT, SPDP or SMPT. The mean derivatization ratios were calculated as described in Materials and Methods.

Table S2. Loss of RIP activity, PNAG activity and cytotoxicity of saporin derivatized with 2-IT, SPDP or SMPT with respect to native saporin.

	-SH/saporin inserted	Loss of RIP activity (Folds)	Loss of PNAG activity (Folds)	Loss of cytotoxicity (Folds)
**2-IT**	1.71	4.68	1.11	4.94
	1.98	6.50	1.18	6.25
	2.52	>7.86	1.30	10.9
**SPDP**	1.91	1.14	1.03	1.45
	2.23	1.23	1.13	2.77
	3.02	1.27	1.40	2.86
**SMPT**	1.20	1.18	1.13	1.97
	1.45	1.41	1.13	2.95
	1.60	1.59	1.30	2.99
